# Improved Rates of Cervical Cancer Screening Among Transmasculine Patients Through Self-Collected Swabs for High-Risk Human Papillomavirus DNA Testing

**DOI:** 10.1089/trgh.2019.0019

**Published:** 2020-03-16

**Authors:** Zil Goldstein, Tyler Martinson, Shruti Ramachandran, Rebecca Lindner, Joshua D. Safer

**Affiliations:** Mount Sinai Center for Transgender Medicine and Surgery, Mount Sinai Health System and Icahn School of Medicine at Mount Sinai, New York, New York.

**Keywords:** transmasculine, female-to-male, transgender health care, cervical cancer, HPV, screening

## Abstract

**Introduction:** Nearly all cervical cancer cases are caused by one of several high-risk strains of the human papillomavirus (hr-HPV). Transmasculine (TM) individuals (persons who have a masculine spectrum gender identity, but were recorded female at birth) have low adherence to standard cervical cancer screening modalities. Introduction of self-collected vaginal swabs for hr-HPV DNA testing may promote initiation and adherence to cervical cancer screening among TM individuals to narrow screening disparities. The purpose of this study was to assess the rate of cervical cancer screening among TM individuals following the introduction of self-collected swabbing for hr-HPV DNA testing in comparison to clinician-administered cervical specimen collection.

**Methods:** Rates of uptake and adherence to cervical cancer screening among TM individuals were assessed before and after the clinical introduction of self-collected swab testing in October 2017. Rates were compared with the rates of cervical cancer screening among cisgender women at a colocated Comprehensive Health Program during the time period of review.

**Results:** Of the 121 TM patients seen for primary care in the 6-month baseline period before the October 2017 introduction of self-collected swabbing for hr-HPV DNA testing, 30 (25%) had cervical cancer screening documented in the electronic medical record. Following the implementation of self-swabbing, of 193 patients, 98 (51%) had a documented cervical cancer screening, a two-fold increase in the rates of adherence to cervical cancer screening (*p*<0.001).

**Conclusion:** Self-collected swab testing for hr-HPV can increase rates of adherence to screening recommendations among an otherwise under-screened population.

## Introduction

Nearly all cervical cancer cases are caused by one of several high-risk strains of the human papillomavirus (hr-HPV), leading to considerable morbidity and mortality in individuals with a cervix.^1^ To date, there is a paucity of research surrounding cervical cancer screening in transmasculine (TM) individuals (persons with masculine spectrum gender identity but recorded female sex at birth) who retain cervix and uterus when compared to cisgender women (persons with feminine spectrum gender identity recorded female sex at birth).

Papanicolaou (Pap) cytologic testing is the recommended screening modality for cervical abnormalities in individuals with a cervix from 21 to 65 years of age, with screening every 3 years if Pap cytology test results are normal.^2^ Although cytology alone and hr-HPV co-testing remain the predominant screening options recommended in clinical guidelines in the United States,^3,4^ clinician-collected cervical swabs for primary hr-HPV screening is approved by the U.S. Food and Drug Administration as an alternative strategy to cytology-based cervical cancer screening methods. Clinician-collected cervical swab has a sensitivity for high-grade cervical squamous intraepithelial lesions (SILs) and invasive cervical cancer that is parallel or superior to that of a Pap smear.^5–8^ However, when validated with transgender men, vaginal self-swabs for HPV have been found to be 71.5% as sensitive as provider-collected swabs.^9^

Despite myths that TM individuals are at low risk for sexually transmitted infections, including HPV and associated cervical abnormalities,^10,11^ recent research suggests that TM patients experience the same level of risk relative to cisgender women for hr-HPV infection and associated progression of cervical abnormalities.^12–14^

The majority (>80%) of TM individuals do not undergo gender-affirming genital surgery or removal of reproductive tract organs, and therefore retain a cervix.^15–17^ However, available data precede widespread insurance coverage for these procedures, and the number of TM individuals who retain their uterus and cervix may change in the future. Routine preventive health screening including screening for cervical cancer, like for cisgender women, is recommended for TM patients who retain their cervix.^9^ Nationally, however, as many as one-third of all TM patients are not up-to-date with screening per U.S. clinical guidelines and TM individuals are more likely to have never undergone cervical cancer screening.^9,18,19^

Among cisgender women, self-collected vaginal swabbing for hr-HPV testing as a primary method for cervical cancer screening has been found to be more acceptable than cervical cytological screening.^20,21^ Self-collection has led to improved rates of adherence to specified American College of Obstetricians and Gynecologists (ACOG) and Centers for Disease Control and Prevention (CDC) screening recommendations among historically under-screened women, suggesting that it could be used as a viable and less invasive screening option.^22–24^ The practice of self-collected swabbing involves the use of a swab by the individual patient to collect a specimen from the patient's own vaginal canal, without the use of a speculum. Introduction of self-collected vaginal swabs for hr-HPV testing and cervical cancer screening for TM patients with retained female reproductive tract organs may promote increased cervical cancer screening and narrow screening disparities if offered after a traditional speculum exam is refused.^9^

Although past research has confirmed the general acceptability of self-collected hr-HPV swabs over clinician-collected hr-HPV or Pap tests for TM individuals in the clinical setting,^25^ to date there are no reports contrasting self- and clinician-collected swabs for hr-HPV testing in TM patients with respect to rates of uptake and adherence to preventive screening protocols.

The purpose of this study was to assess rates of cervical cancer screening uptake and adherence among TM individuals before and after the clinic-wide introduction of a self-collected swab testing intervention. The self-swab option was offered once a traditional speculum exam with cytology collection was refused in patients ages 21–30 and as a primary screening modality in those over 30 with a history of no abnormal Pap testing.

## Methods

### Design and procedures

The study was conducted at the Center for Transgender Medicine and Surgery (CTMS) at Mount Sinai in New York City as part of an ongoing quality improvement project. Approval for publication was obtained from the Institutional Review Board at Mount Sinai.

Eligibility for the study was determined by both patient interview and medical records. Those TM individuals ages 21–30 years who could not recall or document cervical cancer screening within the past 2–3 years with a history of no screening or negative screening along with those ages 30–65 years who could not recall or document screening within the past 5 years were considered eligible if they refused screening with a traditional speculum exam.

The intervention consisted of medical providers offering self-swabs for HPV once a conventional speculum exam for cytology collection was declined. This process included discussion of follow-up options for cytology with hr-HPV screening. Providers instructed those declining conventional screening to insert a cotton-tipped swab into the vagina as far as comfortable, move it in a circular motion, and withdraw the swab. The swab was then vigorously agitated in a conventional ThinPrep™ preparation and sent to the Mount Sinai Health System clinical laboratory for processing.

Rates of uptake and adherence to cervical cancer screening among TM individuals were assessed before and after the implementation of a self-collected swab testing intervention, introduced clinic-wide in October 2017. The baseline historical rate of cervical cancer screening was extracted from an electronic medical record through retrospective chart review of the TM patients who retained a cervix and attended at least one primary care visit from January 2017 through June 2017. Cervical cancer screening rates among TM patients with a cervix and who attended primary care appointments from April 2018 through September 2018, after introduction of the intervention, were determined. Both rates were compared with those among cisgender women at a colocated Comprehensive Health Program during the time period of review. These periods were chosen to avoid provider biases either while the intervention was under discussion and some providers may have adopted early, or heightened sensitivity to the need for cervical cancer screening immediately after implementing the new screening tool.

A Welch two sample *t*-test was completed with R to confirm the statistical significance of the difference between hr-HPV testing coverage in the baseline group and the rate of screening among the group examined after the self-swab intervention was implemented and clinically normalized.

#### Sample size

A total of 394 TM patients received primary care at the CTMS at Mount Sinai during the period studied. Of those patients, 80 had undergone hysterectomy before the study, meaning that there were 314 who required cervical cancer surveillance. The baseline rate of cervical cancer screening was determined through retrospective chart review of the 121 TM patients who retained a cervix and attended at least one primary care visit during the 6-month period from January 2017 through June 2017. This retrospective review addressed all documentation in the patient's chart where a cervical cancer screen would be documented, including progress note free text.

Following the introduction of the self-swab protocol, the intervention cervical cancer screening rate among TM patients was determined from a review of the 193 TM patients with a cervix who presented for primary care appointments during the 6-month period from April 2018 through September 2018.

Among the 314 eligible TM patients, 73 had medical appointments during both the historical and intervention periods. Of the 73, 16 were screened only during the baseline period, and 34 were screened only during the intervention period. The remaining 15 of the 73 patients had cervical cancer screening done in both the pre- and postintervention periods. Without the 73 patients seen in both periods, there were 241 unique TM patients whose appointments did not overlap between the baseline and sample time periods and whose experiences could be cleanly compared.

## Results and Data Analysis

### Characteristics of the study sample

#### Demographics

The patient sample assessed had a mean age of 32 years (range 21–62 years). Of the sample, 58% identified as White, 21% as Black or African American, 12% as Hispanic/Latinx, and 6% as another race or ethnicity. There were 4% unknown or unreported.

The most common terms patients used to describe their gender identities were transgender man (91% of patients) and nonbinary (recorded female at birth, 9%). The patients reported the following sexual orientations: 25% heterosexual, 11% homosexual, 7% bisexual, 20% queer, and 18% some other sexual orientation. There were 19% unknown or unreported. The patients reported gender identities of their sexual partners as predominantly cisgender women (69%), followed by cisgender men (23%), transgender men (16%), and transgender women (16%).

The majority of the sample (95%) reported ever using masculinizing hormone replacement therapy (e.g., testosterone), with 24% of those patients reporting that they had been taking masculinizing hormones for at least 5 years. Seventy-five percent of patients had commercial insurance coverage, while 23% had Medicaid/Medicare, and 2% were uninsured (Table 1).

**Table 1.** Descriptive Characteristics of Transmasculine Sample (*N*=394)

Sociodemographics	Mean	SD
Age, continuous		
Range: 21–62 years	32	9.4

	N	%
Race/Ethnicity		
American Indian or Alaska Native	1	0.3
Asian	15	3.8
Native Hawaiian or Pacific Islander	0	0.0
Black or African American	84	21.3
White	229	58.1
Other	48	12.2
Unknown or not reported	17	4.3
Hispanic/Latinx		
Hispanic or Latinx	46	11.7
Not Hispanic or Latinx	239	60.7
Unknown or not reported	109	27.7
Gender identity		
Transgender man	359	91.1
Nonbinary, recorded female at birth	35	8.9
Insurance		
No health insurance	7	1.8
Public insurance (Medicaid, Medicare)	92	23.4
Private, school or work insurance	295	74.9
Sexual orientation and sexual partnering		
Sexual orientation		
Gay/homosexual/same-gender attracted	43	10.9
Straight/heterosexual	99	25.1
Bisexual	27	6.9
Queer	80	20.3
Other	70	17.8
Unknown	75	19.0
Gender of sexual partners		
Cisgender man	92	23.4
Cisgender woman	272	69.0
Transgender man	61	15.5
Transgender woman	39	9.9
Other	39	9.9
Medical gender affirmation		
Hormone use		
Yes	376	95.4
No	18	4.6
Time on hormones	*n*=376	
Less than 6 months	28	7.4
6 months to less than 12 months	21	5.6
12 months to less than 3 years	132	35.1
3 years to less than 5 years	21	5.6
5 years or more	90	23.9
Unknown	84	22.3

SD, standard deviation.

There were no statistically significant intergroup differences in age, race/ethnicity, gender identity, or sexual orientation between the baseline and the intervention groups (Table 2).

**Table 2.** Descriptive Characteristics of Baseline Versus Intervention Groups (*N*=314)

	Baseline (N=l21)		Intervention (N=l93)		p
	Mean	SD	Mean	SD	
Age, continuous					0.08
Range: 21–62 years	32.06	9.92	30.90	8.07	
	*N*	%	*N*	%	
Race/Ethnicity					0.11
Asian	6	5.0	7	3.6	
Black or African American	24	19.8	43	22.3	
White	64	52.9	119	61.7	
Other/Unknown	27	22.3	24	12.4	
Hispanic/Latinx					0.24
Hispanic or Latinx	13	10.7	20	10.4	
Not Hispanic or Latinx	65	53.7	121	62.7	
Unknown or not reported	43	35.5	52	26.9	
Gender identity					0.68
Transgender man	109	90	171	88.6	
Nonbinary, recorded female at birth	12	9.9	22	11.4	
Sexual orientation					0.82
Gay, lesbian, or homosexual	13	10.7	19	9.8	
Bisexual	7	5.8	16	8.3	
Straight/heterosexual	28	23.1	37	19.2	
Queer	24	19.8	44	22.8	
Other	22	18.2	40	20.7	
Unknown/declined to answer	27	22.3	37	19.2	

### Self-swab hr-HPV DNA screening improved utilization and cervical cancer screening status

#### Utilization of Pap test versus self-swab

**FIG. 1.**
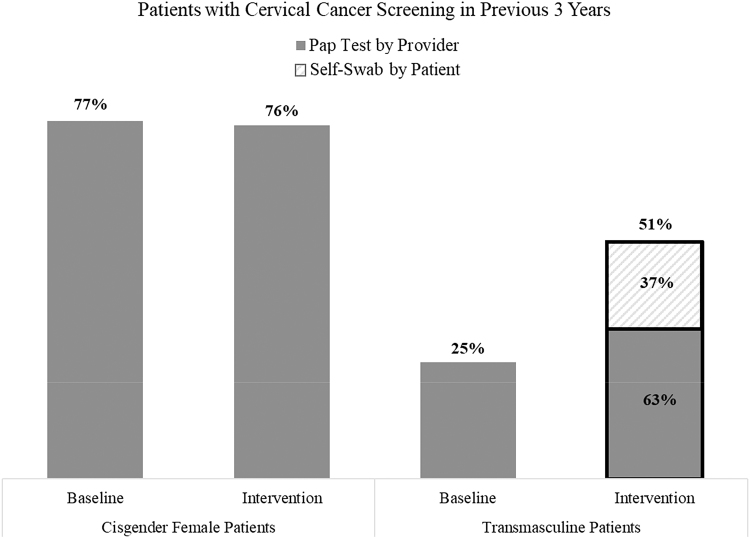
Patients with cervical cancer screening in previous 3 years. Of the 121 TM patients seen for primary care in the 6-month baseline period before the intervention, 30 (25%) had cervical cancer screening documented in their chart. After the October 2017 implementation of the clinic-wide intervention encouraging clinicians to offer self-collected swabs for hr-HPV testing, of the 193 TM patients seen for primary care from April 1, 2018 to September 30, 2018, 98 (51%) had a documented cervical cancer screening in their chart, indicating a two-fold increase in the proportion of TM individuals adhering to cervical cancer screening recommendations. Among the 98 patients screened for cervical cancer in the intervention period, 36 screened with self-swab, representing the majority of the increase in screenings. The rate of cervical cancer screening among cisgender women at a colocated Comprehensive Health Program remained constant, with 77% coverage during the baseline period and 76% following the self-swab intervention. hr-HPV, human papillomavirus; TM, transmasculine.

Of the 121 TM patients seen for primary care during the baseline period, 30 (25%) had cervical cancer screening following the recommended timeline documented in their chart. All were clinician-administered Pap tests (Fig. 1). The majority, 19 (63%), included hr-HPV co-testing (hr-HPV 16/18 or HPV 31/33/35/39/45/51/52/56/58/59/66/68). Of the 30 cervical cancer screening procedures 15 (50%) occurred specifically within the 6-month baseline period. The rate of adherence to cervical cancer screening protocols among cisgender women at a colocated primary care and infectious disease clinic was 77% during the same period.

Following the introduction of self-collected swabs for hr-HPV, 98 (51%) of the 193 TM patients seen for primary care had a documented cervical cancer screening in their chart, indicating a two-fold increase in adherence to cervical cancer screening recommendations among TM patients (Fig. 1). The rate of cervical cancer screening among cisgender women during this intervention period was 76%. Of the 98 screening procedures, 38 (39%) involved hr-HPV testing exclusively, with 36 of those 38 (95%) specimens collected from self-swab. Further, among the 98 procedures, 51 (52%) took place during the intervention period. The majority of those 51 (28, 55%) were completed via self-swab while 23 (45%) of the 51 were Pap tests performed by a clinician.

#### Baseline Pap test SIL detection and hr-HPV prevalence

All samples collected from the Pap tests performed by clinicians during the baseline period were determined to be satisfactory for evaluation. All testing in the baseline period was negative for either intraepithelial lesions or malignancy. Of the 15 specimens co-tested for hr-HPV during the baseline period, 4 (27%) were positive for HPV 31/33/35/39/45/51/52/56/58/59/66/68.

#### Intervention Pap test SIL detection and hr-HPV prevalence

Of the 51 cervical cancer screening procedures completed during the 6-month intervention period, 4 (8%) were determined to be unsatisfactory for evaluation with indeterminate results. All 4 unsatisfactory specimens were clinician-collected cytology tests, comprising 17% of the 23 clinician-collected tests. Of the remaining clinician-collected cytology specimens, 1 (5%) detected SILs requiring follow-up, and 1 (8%) of the 13 specimens co-tested for hr-HPV tested positive for HPV genotypes 31/33/35/39/45/51/52/56/58/59/66/68.

All hr-HPV self-swabs were satisfactory for laboratory analysis. HPV genotypes 31/33/35/39/45/51/52/56/58/59/66/68 were detected in 2 (7%) of the self-swab samples. All abnormal results were referred for colposcopy, but adherence data and results were not available at the time of the chart review.

### Statistical analysis

A Welch two sample *t*-test was completed with R and confirmed the statistical significance of the difference between the 25% cervical cancer screening in the baseline period and the 51% rate of screening in the intervention period (*t*=−4.8624, df=281.71, *p*<0.001, 95% CI: −0.36 to −0.15).

## Discussion

To our knowledge, this is the first assessment of increased hr-HPV testing and cervical cancer screening coverage among TM patients receiving primary care in an urban center following the introduction of self-collected swabbing as the primary hr-HPV DNA testing strategy. The increase is consistent with literature reporting self-swab to be a preferred method of cervical cancer screening for TM individuals.^22^ Self-collected swabs for hr-HPV DNA testing improved rates of participation and engagement in cervical cancer screening among TM patients and, consequently, detection of hr-HPV.

During the baseline period, we were able to successfully screen only 25% of patients. While we were able to screen 51% of patients in the intervention period with either provider- or patient-collected specimens, the lower sensitivity of hr-HPV self-swabs in TM would mean that 44% of primary care patients received an accurate screening test, still a significant improvement over the preintervention period. However, the lower sensitivity of self-swabs among TM individuals calls into question the proper screening interval with the use of primary hr-HPV screening in this population. There are not enough data available on TM individuals and cervical cancer to make a recommendation at this time.

The observed increase in cervical cancer screening among TM patients after the introduction of self-swabbing, from 25% in 2017 to 51% in 2018, is still lower than the 76% screening coverage of cisgender women in the colocated Comprehensive Health Program, and the national rate of 85% of insured cisgender women who are historically up-to-date with cervical cancer screening.^26^ Previous studies have also found that TM individuals have lower lifetime rates of Pap test adherence and are significantly less likely to be current with cervical cancer screening. For example, one study at a similar center showed 74% adherence to screening among cisgender female patients and 64% among TM patients.^19^ Another showed 84% for cisgender female patients and 69% among TM patients.^18^ Notably, the rates of adherence exceed that of the rates accomplished here and suggest additional examination of barriers impeding access to cervical cancer screening for TM individuals at Mount Sinai.

Transgender and gender non-binary (TGNB) individuals are the subject of very little focused health research.^27^ The literature documents multiple barriers to consistent engagement with cervical cancer screening for TM individuals. The biggest barrier is the lack of access to affordable, affirming, and quality care.^28^ Barriers to health care include those that are direct—economic instability, unemployment, being victims of violence, and lack of health insurance^28–34^—along with those that are indirect (e.g., continue even as access to health care increases), such as the paucity of knowledgeable health care providers.^31–43^ Despite several clinical practice guidelines,^44–47^ scarcity of competent clinicians continues to represent the greatest barrier reported by TGNB individuals.^28,29,31,34–37^

Nationally, TGNB individuals are significantly less likely to have health insurance, with a 14% un-insurance rate,^48^ and are likelier to have unmet routine medical care needs due to cost, compared to cisgender individuals.^49,50^ With broad coverage for transgender care, New York state serves as an excellent setting to examine disparities in care among TGNB individuals beyond lack of coverage for care. In states without expanded Medicaid coverage, access to cervical cancer screening is still limited for the uninsured or underinsured. Only 2% of the TM patients in our sample were uninsured. TGNB-competent care training should include discussion of the unique primary care and preventive screening service needs of TGNB patients to promote the ongoing development of clinical recommendations from evidence-based observational and intervention studies throughout all levels of care.^51^

This study utilized a convenience sample, a weakness for generalizability. We examined a patient sample of TM individuals already well-engaged in primary care at an academic medical center specializing in TGNB health care who may have been more motivated than average to engage in general medical care and follow clinician recommendations to adhere to preventive screening procedures. Relatedly, our study likely suffered clinician bias in that introduction of the self-swab protocol may have increased clinician vigilance with regard to screening recommendations and surveillance. Clinicians were not aware that there would be a change in protocol during the baseline period. The stability of cervical cancer screening rates among the cisgender women after increased surveillance suggests that the effect was modest. Given the importance of provider recommendation for motivating cisgender people to undergo cancer screening, the role of the relationship between provider and patients should be further investigated in TGNB samples.^26,52–54^

## Conclusion

Self-collected swab testing to screen for hr-HPV can increase rates of uptake and adherence to screening recommendations among an under-screened and underserved patient population. Self-collected swab testing could prove a key element to shrink screening disparities and progress toward more optimal care for TGNB individuals. Future research should continue to explore less invasive, patient-empowering, and gender-affirming screening strategies within primary care for TGNB individuals and focus on increasing the sensitivity of self-swab specimens in TM individuals.

## Abbreviations Used

ACOG: American College of Obstetricians and Gynecologists
CDC: Centers for Disease Control and Prevention
hr-HPV: human papillomavirus
Pap: papanicolaou
SD: standard deviation
SIL: squamous intraepithelial lesion
TGNB: transgender and gender non-binary
TM: transmasculine
